# Crystal structure of (2-{[3,5-bis­(1,1-di­methyl­eth­yl)-4-hy­droxy­phen­yl](5-methyl-2*H*-pyrrol-2-yl­idene)meth­yl}-5-methyl-1*H*-pyrrolido-κ^2^ *N*,*N*′)di­fluoridoboron

**DOI:** 10.1107/S2056989015015789

**Published:** 2015-08-29

**Authors:** Yukio Morimoto, Keizo Ogawa, Yoshihiro Uto, Hideko Nagasawa, Hitoshi Hori

**Affiliations:** a Research Reactor Institute, Kyoto University, 2-1 Asashiro-Nishi, Kumatori, Osaka 590-0494, Japan; b Nihon University Junior College, 7-24-1 Narashinodai, Funabashi, Chiba 274-8501, Japan; c Department of Engineering, The University of Tokushima, Minami-Josanjima, Tokushima 770-8506, Japan; d Laboratory of Pharmaceutical & Medicinal Chemistry, Gifu Pharmaceutical University, 1-25-4 Daigakunishi, Gifu, 501-1196, Japan; e Niigata University of Pharmacy and Applied Life Sciences, 265-1 Higashijima, Akiha-ku, Niigata 956-8603, Japan

**Keywords:** crystal structure, boron tracedrug, boron neutron capture therapy (BNCT)

## Abstract

The title compound, C_25_H_31_BF_2_N_2_O, is a potential boron tracedrug in boron neutron capture therapy (BNCT), in which the B atom adopts a distorted BN_2_F_2_ tetra­hedral geometry: it is soluble in dimethyl sulfoxide, di­methyl­formamide and methanol. The pyrrolyl­idene­methyl­pyrrole triple fused ring system is almost planar (r.m.s. deviation = 0.031 Å) and subtends a dihedral angle of 47.09 (5)° with the plane of the pendant phenol ring. The phenol –OH group is blocked from forming hydrogen bonds by the adjacent bulky *tert*-butyl groups. In the crystal, inversion dimers linked by pairs of very weak C—H⋯F inter­actions generate *R*_2_^2^(22) loops.

## Related literature   

For background to tracer compounds for BNCT, see: Hori *et al.* (2010 ▸, 2012 ▸). For further synthetic details, see: Nakata *et al.* (2011 ▸).
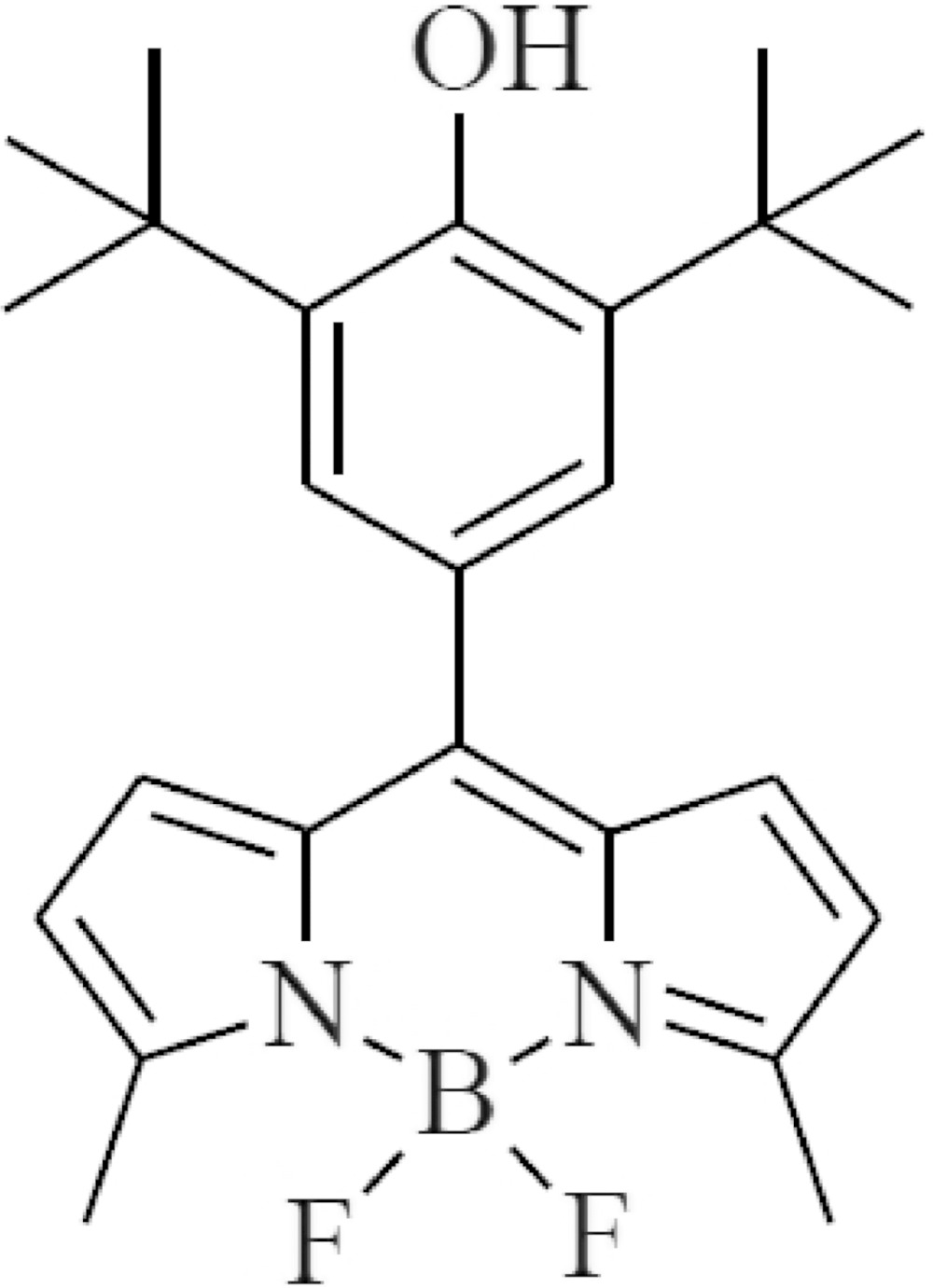


## Experimental   

### Crystal data  

C_25_H_31_BF_2_N_2_O
*M* *_r_* = 424.34
Triclinic, 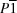

*a* = 9.2518 (2) Å
*b* = 10.0975 (2) Å
*c* = 12.5142 (3) Å
α = 79.364 (6)°
β = 89.613 (6)°
γ = 83.367 (6)°
*V* = 1141.18 (5) Å^3^
*Z* = 2
Cu *K*α radiation
μ = 0.69 mm^−1^
*T* = 296 K
0.16 × 0.08 × 0.04 mm

### Data collection  

Rigaku R-AXIS RAPID diffractometer
Absorption correction: multi-scan (*ABSCOR*; Rigaku, 1995 ▸) *T*_min_ = 0.749, *T*_max_ = 0.973
13606 measured reflections
4044 independent reflections
3644 reflections with *F* ^2^ > 2.0σ(*F* ^2^)
*R*_int_ = 0.026

### Refinement  

*R*[*F* ^2^ > 2σ(*F* ^2^)] = 0.040
*wR*(*F* ^2^) = 0.115
*S* = 1.09
4044 reflections
289 parameters
H-atom parameters constrained
Δρ_max_ = 0.29 e Å^−3^
Δρ_min_ = −0.20 e Å^−3^

### 

Data collection: *RAPID-AUTO* (Rigaku, 2011 ▸); cell refinement: *RAPID-AUTO*; data reduction: *RAPID-AUTO*; program(s) used to solve structure: *Il Milione* (Burla *et al.*, 2007 ▸); program(s) used to refine structure: *SHELXL97* (Sheldrick, 2008 ▸); molecular graphics: *CrystalStructure* (Rigaku, 2011 ▸); software used to prepare material for publication: *CrystalStructure*.

## Supplementary Material

Crystal structure: contains datablock(s) global, I. DOI: 10.1107/S2056989015015789/hb7487sup1.cif

Structure factors: contains datablock(s) I. DOI: 10.1107/S2056989015015789/hb7487Isup2.hkl

**Fig. 1.**
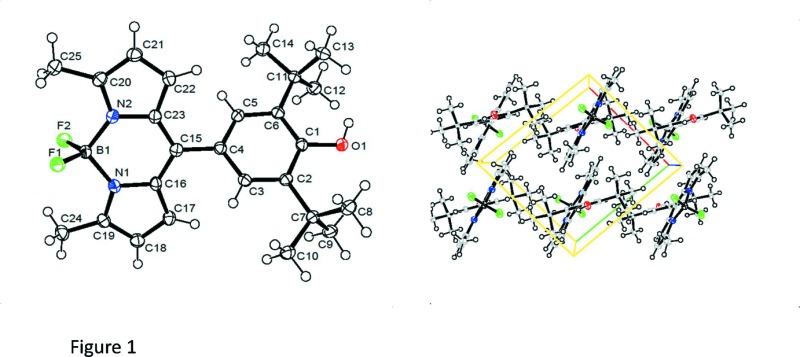
Molecular structure of the compound. Displacement ellipsoids are shown at the 50% probability level. H atoms are depicted as small spheres of arbitrary radius.

. DOI: 10.1107/S2056989015015789/hb7487fig1.tif
Mol­ecular structure of the compound. Displacement ellipsoids are shown at the 50% probability level. H atoms are depicted as small spheres of arbitrary radius.

CCDC reference: 1420063

Additional supporting information:  crystallographic information; 3D view; checkCIF report

## Figures and Tables

**Table 1:** Hydrogen-bond geometry (, )

*D*H*A*	*D*H	H*A*	*D* *A*	*D*H*A*
C12H12*C*F2^i^	0.96	2.54	3.4464(18)	158

Symmetry code: (i) 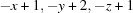.

## supplementary crystallographic information

### S1. Chemical context

\ For the synthesis of 2-{[3,5-bis­(1,1-di­methyl­ethyl)-4-hy­droxy­phenyl](5-methyl-2*H*-pyrrol-2-\ yl­idene)methyl}-5-methyl-1*H*-pyrrolido-κ^2^*N*,*N*']\ difluoridoboron see: Nakata, *et al.* (2011). The compound: CAS Registry Number 1415304-92-5. The ring compound binding BF_2_ is a contracted form given in IUPAC.

### S2. Structural commentary

The traceability of boron tracedrugs is based on the neutron capture activity of the stable isotope boron-10 embedded in the drug. Thus, newly designed boron tracedrugs would be novel pharmaceuticals, the structures of which would always include natural boron (B^11^, 80.4%; B^10^, 19.6%), as tracers, embedded deeply in their skeletons or scaffolds. The compound is used at a cancer therapy by an irradiation of neutron, since B^10^ atom generates a high energy alpha-line within a cancer cell by fission of the atom. The compound is required to keep a suitable three-dimensional structure until reaching cancer cell via intra­venous injection and an irradiation of neutron. Our group has developed boron tracedrugs in use of boron-neutron reaction (Hori, *et al.* 2010, 2012), in this study a suitable compound has been reported and presents that two bulky rings tilt each other to avoid steric hindrance.

### S3. Synthesis and crystallization

Crystals were obtained from methanol solvent at room temperature by slow evaporation. Crystal structure has no present of solvent molecule. The synthesis of the title compound was decribed by Nakata *et al.* (2011). The compound: CAS Registry Number 1415304-92-5. The ring compound binding BF_2_ is a contracted form given in IUPAC.

### S4. Refinement

All hydrogen atoms were placed in the calculated positions and constrained their parent atoms with a C—H distances of 0.95 Å (aromatic) and 0.99 Å (methyl­ene) and with *U*iso(H) = 1.2*U*eq(C), and 0.98 Å for CH3 [*U*iso(H)= 1.5*U*eq(C)].

### Crystal data

**Table:**

C_25_H_31_BF_2_N_2_O	*Z* = 2
*M**_r_* = 424.34	*F*(000) = 452.00
Triclinic, *P*1	*D*_x_ = 1.235 Mg m^−^^3^
Hall symbol: -P 1	Cu *K*α radiation, λ = 1.54187 Å
*a* = 9.2518 (2) Å	Cell parameters from 11333 reflections
*b* = 10.0975 (2) Å	θ = 3.6–68.2°
*c* = 12.5142 (3) Å	µ = 0.69 mm^−^^1^
α = 79.364 (6)°	*T* = 296 K
β = 89.613 (6)°	Platelet, orange
γ = 83.367 (6)°	0.16 × 0.08 × 0.04 mm
*V* = 1141.18 (5) Å^3^	

### Data collection

**Table:**

Rigaku R-AXIS RAPID diffractometer	3644 reflections with *F*^2^ > 2.0σ(*F*^2^)
Detector resolution: 10.000 pixels mm^-1^	*R*_int_ = 0.026
ω scans	θ_max_ = 67.5°, θ_min_ = 3.6°
Absorption correction: multi-scan (*ABSCOR*; Rigaku, 1995)	*h* = −10→10
*T*_min_ = 0.749, *T*_max_ = 0.973	*k* = −12→12
13606 measured reflections	*l* = −14→14
4044 independent reflections	

### Refinement

**Table:**

Refinement on *F*^2^	Secondary atom site location: difference Fourier map
*R*[*F*^2^ > 2σ(*F*^2^)] = 0.040	Hydrogen site location: inferred from neighbouring sites
*wR*(*F*^2^) = 0.115	H-atom parameters constrained
*S* = 1.09	*w* = 1/[σ^2^(*F*_o_^2^) + (0.0594*P*)^2^ + 0.4471*P*] where *P* = (*F*_o_^2^ + 2*F*_c_^2^)/3
4044 reflections	(Δ/σ)_max_ < 0.001
289 parameters	Δρ_max_ = 0.29 e Å^−^^3^
0 restraints	Δρ_min_ = −0.20 e Å^−^^3^
Primary atom site location: structure-invariant direct methods	

### Special details

**Table:**

Geometry. ENTER SPECIAL DETAILS OF THE MOLECULAR GEOMETRY
Refinement. Refinement was performed using all reflections. The weighted *R*-factor (*wR*) and goodness of fit (*S*) are based on *F*^2^. *R*-factor (gt) are based on *F*. The threshold expression of *F*^2^ > 2.0 σ(*F*^2^) is used only for calculating *R*-factor (gt).

### Fractional atomic coordinates and isotropic or equivalent isotropic displacement parameters (Å^2^)

**Table:**

	*x*	*y*	*z*	*U*_iso_*/*U*_eq_	
F1	0.16643 (10)	0.79448 (8)	0.09158 (7)	0.0297 (2)	
F2	0.39555 (10)	0.84379 (8)	0.11327 (7)	0.0294 (2)	
O1	0.22265 (12)	0.68344 (10)	0.86928 (8)	0.0231 (2)	
N1	0.30765 (13)	0.67065 (12)	0.24855 (9)	0.0191 (3)	
N2	0.21212 (13)	0.91383 (12)	0.23671 (9)	0.0193 (3)	
C1	0.23318 (15)	0.70635 (14)	0.75831 (11)	0.0181 (3)	
C2	0.18152 (15)	0.60808 (13)	0.70604 (11)	0.0183 (3)	
C3	0.18647 (15)	0.63023 (14)	0.59314 (11)	0.0187 (3)	
C4	0.24376 (15)	0.74233 (13)	0.53267 (11)	0.0185 (3)	
C5	0.29681 (15)	0.83469 (14)	0.58803 (11)	0.0188 (3)	
C6	0.29266 (15)	0.82094 (13)	0.70055 (11)	0.0185 (3)	
C7	0.12345 (16)	0.47938 (14)	0.76987 (11)	0.0203 (3)	
C8	−0.00798 (17)	0.51496 (15)	0.83913 (12)	0.0263 (3)	
C9	0.24586 (17)	0.39358 (14)	0.84310 (12)	0.0252 (3)	
C10	0.07400 (18)	0.38987 (15)	0.69353 (12)	0.0257 (3)	
C11	0.34936 (16)	0.92882 (14)	0.75712 (11)	0.0207 (3)	
C12	0.47337 (17)	0.86627 (15)	0.83888 (12)	0.0264 (3)	
C13	0.22373 (18)	1.00162 (15)	0.81377 (13)	0.0267 (3)	
C14	0.41194 (18)	1.03996 (15)	0.67502 (12)	0.0274 (3)	
C15	0.24904 (15)	0.76195 (14)	0.41279 (11)	0.0189 (3)	
C16	0.30154 (15)	0.65409 (14)	0.36187 (11)	0.0189 (3)	
C17	0.35863 (16)	0.51942 (14)	0.40509 (12)	0.0219 (3)	
C18	0.39704 (17)	0.45610 (15)	0.31931 (12)	0.0241 (3)	
C19	0.36563 (16)	0.55126 (14)	0.22312 (12)	0.0213 (3)	
C20	0.16017 (16)	1.04503 (14)	0.19924 (12)	0.0218 (3)	
C21	0.11602 (16)	1.10733 (15)	0.28709 (12)	0.0235 (3)	
C22	0.14065 (16)	1.01132 (14)	0.38080 (12)	0.0218 (3)	
C23	0.20257 (15)	0.88981 (14)	0.34988 (11)	0.0192 (3)	
C24	0.38939 (18)	0.53166 (16)	0.10909 (12)	0.0259 (3)	
C25	0.15235 (18)	1.10739 (15)	0.08147 (12)	0.0264 (3)	
B1	0.27066 (19)	0.80652 (16)	0.16769 (13)	0.0209 (3)	
H1	0.24213	0.7504	0.8922	0.0277*	
H3	0.15046	0.56837	0.55667	0.0224*	
H5	0.33661	0.90855	0.5481	0.0225*	
H8A	−0.03914	0.43306	0.87955	0.0316*	
H8B	−0.08608	0.56366	0.79254	0.0316*	
H8C	0.01911	0.5704	0.88858	0.0316*	
H9A	0.21123	0.31164	0.88038	0.0302*	
H9B	0.2753	0.44429	0.89533	0.0302*	
H9C	0.32743	0.37128	0.79929	0.0302*	
H10A	−0.002	0.44051	0.64586	0.0308*	
H10B	0.03821	0.31134	0.73579	0.0308*	
H10C	0.15488	0.36182	0.65121	0.0308*	
H12A	0.55293	0.8262	0.80142	0.0317*	
H12B	0.43873	0.79781	0.8935	0.0317*	
H12C	0.50577	0.93581	0.87267	0.0317*	
H13A	0.25886	1.07288	0.84391	0.0320*	
H13B	0.18676	0.93777	0.87091	0.0320*	
H13C	0.14735	1.03955	0.76174	0.0320*	
H14A	0.49141	0.99973	0.63759	0.0329*	
H14B	0.44603	1.10512	0.71286	0.0329*	
H14C	0.33743	1.08438	0.62338	0.0329*	
H17	0.36849	0.48053	0.47844	0.0262*	
H18	0.43675	0.36626	0.32406	0.0289*	
H21	0.0771	1.1973	0.28295	0.0282*	
H22	0.12026	1.02419	0.45129	0.0261*	
H24A	0.43805	0.60446	0.07017	0.0311*	
H24B	0.29725	0.531	0.0745	0.0311*	
H24C	0.44821	0.44688	0.10904	0.0311*	
H25A	0.08056	1.06873	0.04575	0.0316*	
H25B	0.2455	1.09007	0.04932	0.0316*	
H25C	0.12595	1.20352	0.07348	0.0316*	

### Atomic displacement parameters (Å^2^)

**Table:**

	*U*^11^	*U*^22^	*U*^33^	*U*^12^	*U*^13^	*U*^23^
F1	0.0432 (6)	0.0228 (4)	0.0232 (5)	−0.0056 (4)	−0.0130 (4)	−0.0026 (3)
F2	0.0376 (5)	0.0257 (5)	0.0273 (5)	−0.0101 (4)	0.0142 (4)	−0.0075 (4)
O1	0.0345 (6)	0.0206 (5)	0.0158 (5)	−0.0088 (4)	0.0015 (4)	−0.0040 (4)
N1	0.0194 (6)	0.0217 (6)	0.0168 (6)	−0.0031 (5)	0.0001 (5)	−0.0048 (5)
N2	0.0195 (6)	0.0208 (6)	0.0173 (6)	−0.0034 (5)	0.0001 (5)	−0.0018 (5)
C1	0.0189 (7)	0.0192 (7)	0.0155 (7)	0.0005 (5)	0.0001 (5)	−0.0027 (5)
C2	0.0172 (7)	0.0167 (7)	0.0199 (7)	−0.0008 (5)	0.0001 (5)	−0.0018 (5)
C3	0.0194 (7)	0.0183 (7)	0.0190 (7)	−0.0021 (5)	−0.0009 (5)	−0.0051 (5)
C4	0.0182 (7)	0.0189 (7)	0.0183 (7)	−0.0010 (5)	0.0008 (5)	−0.0037 (5)
C5	0.0185 (7)	0.0181 (7)	0.0191 (7)	−0.0022 (5)	0.0014 (5)	−0.0014 (5)
C6	0.0176 (7)	0.0182 (7)	0.0198 (7)	−0.0009 (5)	−0.0003 (5)	−0.0042 (5)
C7	0.0248 (8)	0.0178 (7)	0.0183 (7)	−0.0047 (6)	−0.0001 (6)	−0.0021 (5)
C8	0.0284 (9)	0.0232 (7)	0.0274 (8)	−0.0073 (6)	0.0057 (6)	−0.0021 (6)
C9	0.0321 (9)	0.0182 (7)	0.0242 (8)	−0.0032 (6)	−0.0039 (6)	−0.0008 (6)
C10	0.0325 (9)	0.0218 (7)	0.0239 (8)	−0.0099 (6)	−0.0005 (6)	−0.0027 (6)
C11	0.0258 (8)	0.0186 (7)	0.0188 (7)	−0.0054 (6)	0.0004 (6)	−0.0045 (6)
C12	0.0302 (9)	0.0247 (8)	0.0259 (8)	−0.0080 (6)	−0.0040 (6)	−0.0058 (6)
C13	0.0348 (9)	0.0199 (7)	0.0274 (8)	−0.0050 (6)	0.0054 (7)	−0.0085 (6)
C14	0.0363 (9)	0.0245 (8)	0.0241 (8)	−0.0133 (7)	0.0010 (7)	−0.0056 (6)
C15	0.0160 (7)	0.0217 (7)	0.0195 (7)	−0.0055 (5)	−0.0001 (5)	−0.0034 (6)
C16	0.0188 (7)	0.0234 (7)	0.0151 (7)	−0.0051 (6)	−0.0002 (5)	−0.0036 (5)
C17	0.0218 (8)	0.0241 (7)	0.0187 (7)	−0.0011 (6)	−0.0014 (6)	−0.0025 (6)
C18	0.0253 (8)	0.0211 (7)	0.0248 (8)	0.0027 (6)	−0.0003 (6)	−0.0048 (6)
C19	0.0194 (8)	0.0239 (7)	0.0212 (7)	−0.0015 (6)	0.0006 (6)	−0.0065 (6)
C20	0.0196 (8)	0.0220 (7)	0.0225 (7)	−0.0029 (6)	−0.0013 (6)	−0.0005 (6)
C21	0.0239 (8)	0.0198 (7)	0.0257 (8)	0.0010 (6)	0.0000 (6)	−0.0034 (6)
C22	0.0219 (8)	0.0245 (7)	0.0193 (7)	−0.0019 (6)	0.0017 (6)	−0.0053 (6)
C23	0.0190 (7)	0.0220 (7)	0.0170 (7)	−0.0044 (6)	0.0007 (5)	−0.0035 (5)
C24	0.0285 (8)	0.0277 (8)	0.0219 (8)	0.0006 (6)	0.0021 (6)	−0.0078 (6)
C25	0.0308 (9)	0.0245 (8)	0.0218 (8)	−0.0009 (6)	−0.0013 (6)	−0.0005 (6)
B1	0.0268 (9)	0.0209 (8)	0.0159 (8)	−0.0063 (7)	0.0005 (6)	−0.0034 (6)

### Geometric parameters (Å, º)

**Table:**

F1—B1	1.391 (2)	C22—C23	1.412 (2)
F2—B1	1.391 (2)	O1—H1	0.820
O1—C1	1.3698 (17)	C3—H3	0.930
N1—C16	1.3985 (18)	C5—H5	0.930
N1—C19	1.3536 (19)	C8—H8A	0.960
N1—B1	1.5515 (18)	C8—H8B	0.960
N2—C20	1.3537 (17)	C8—H8C	0.960
N2—C23	1.3960 (18)	C9—H9A	0.960
N2—B1	1.555 (2)	C9—H9B	0.960
C1—C2	1.412 (2)	C9—H9C	0.960
C1—C6	1.4132 (19)	C10—H10A	0.960
C2—C3	1.3904 (19)	C10—H10B	0.960
C2—C7	1.5453 (19)	C10—H10C	0.960
C3—C4	1.3971 (19)	C12—H12A	0.960
C4—C5	1.393 (2)	C12—H12B	0.960
C4—C15	1.4778 (19)	C12—H12C	0.960
C5—C6	1.3899 (19)	C13—H13A	0.960
C6—C11	1.544 (2)	C13—H13B	0.960
C7—C8	1.536 (2)	C13—H13C	0.960
C7—C9	1.5386 (19)	C14—H14A	0.960
C7—C10	1.536 (2)	C14—H14B	0.960
C11—C12	1.540 (2)	C14—H14C	0.960
C11—C13	1.542 (2)	C17—H17	0.930
C11—C14	1.538 (2)	C18—H18	0.930
C15—C16	1.399 (2)	C21—H21	0.930
C15—C23	1.4045 (18)	C22—H22	0.930
C16—C17	1.4096 (18)	C24—H24A	0.960
C17—C18	1.372 (2)	C24—H24B	0.960
C18—C19	1.4036 (19)	C24—H24C	0.960
C19—C24	1.488 (2)	C25—H25A	0.960
C20—C21	1.400 (2)	C25—H25B	0.960
C20—C25	1.491 (2)	C25—H25C	0.960
C21—C22	1.3784 (19)		
			
C16—N1—C19	107.94 (11)	C4—C3—H3	118.911
C16—N1—B1	125.50 (12)	C4—C5—H5	118.675
C19—N1—B1	126.23 (12)	C6—C5—H5	118.675
C20—N2—C23	107.87 (12)	C7—C8—H8A	109.472
C20—N2—B1	126.91 (11)	C7—C8—H8B	109.471
C23—N2—B1	125.21 (11)	C7—C8—H8C	109.472
O1—C1—C2	115.70 (12)	H8A—C8—H8B	109.462
O1—C1—C6	121.79 (13)	H8A—C8—H8C	109.480
C2—C1—C6	122.51 (13)	H8B—C8—H8C	109.471
C1—C2—C3	117.24 (12)	C7—C9—H9A	109.472
C1—C2—C7	122.35 (12)	C7—C9—H9B	109.469
C3—C2—C7	120.40 (13)	C7—C9—H9C	109.475
C2—C3—C4	122.18 (14)	H9A—C9—H9B	109.473
C3—C4—C5	118.46 (13)	H9A—C9—H9C	109.469
C3—C4—C15	120.80 (13)	H9B—C9—H9C	109.470
C5—C4—C15	120.73 (12)	C7—C10—H10A	109.472
C4—C5—C6	122.65 (13)	C7—C10—H10B	109.473
C1—C6—C5	116.91 (13)	C7—C10—H10C	109.472
C1—C6—C11	122.82 (12)	H10A—C10—H10B	109.472
C5—C6—C11	120.27 (12)	H10A—C10—H10C	109.464
C2—C7—C8	111.62 (11)	H10B—C10—H10C	109.474
C2—C7—C9	109.37 (12)	C11—C12—H12A	109.467
C2—C7—C10	111.74 (11)	C11—C12—H12B	109.473
C8—C7—C9	109.89 (11)	C11—C12—H12C	109.474
C8—C7—C10	107.04 (13)	H12A—C12—H12B	109.472
C9—C7—C10	107.06 (12)	H12A—C12—H12C	109.466
C6—C11—C12	111.63 (11)	H12B—C12—H12C	109.475
C6—C11—C13	110.32 (12)	C11—C13—H13A	109.473
C6—C11—C14	111.71 (12)	C11—C13—H13B	109.475
C12—C11—C13	110.63 (12)	C11—C13—H13C	109.468
C12—C11—C14	106.21 (12)	H13A—C13—H13B	109.476
C13—C11—C14	106.14 (11)	H13A—C13—H13C	109.463
C4—C15—C16	120.26 (11)	H13B—C13—H13C	109.472
C4—C15—C23	119.79 (13)	C11—C14—H14A	109.471
C16—C15—C23	119.95 (12)	C11—C14—H14B	109.473
N1—C16—C15	121.13 (11)	C11—C14—H14C	109.466
N1—C16—C17	107.58 (13)	H14A—C14—H14B	109.468
C15—C16—C17	131.26 (13)	H14A—C14—H14C	109.475
C16—C17—C18	107.60 (12)	H14B—C14—H14C	109.473
C17—C18—C19	107.72 (13)	C16—C17—H17	126.206
N1—C19—C18	109.16 (13)	C18—C17—H17	126.198
N1—C19—C24	122.81 (12)	C17—C18—H18	126.137
C18—C19—C24	128.03 (13)	C19—C18—H18	126.139
N2—C20—C21	109.37 (12)	C20—C21—H21	126.155
N2—C20—C25	123.17 (14)	C22—C21—H21	126.156
C21—C20—C25	127.45 (12)	C21—C22—H22	126.369
C20—C21—C22	107.69 (13)	C23—C22—H22	126.370
C21—C22—C23	107.26 (13)	C19—C24—H24A	109.466
N2—C23—C15	121.42 (13)	C19—C24—H24B	109.471
N2—C23—C22	107.79 (11)	C19—C24—H24C	109.467
C15—C23—C22	130.78 (13)	H24A—C24—H24B	109.474
F1—B1—F2	108.79 (12)	H24A—C24—H24C	109.473
F1—B1—N1	110.89 (13)	H24B—C24—H24C	109.476
F1—B1—N2	110.50 (12)	C20—C25—H25A	109.472
F2—B1—N1	109.74 (12)	C20—C25—H25B	109.473
F2—B1—N2	110.35 (13)	C20—C25—H25C	109.478
N1—B1—N2	106.56 (11)	H25A—C25—H25B	109.464
C1—O1—H1	109.476	H25A—C25—H25C	109.468
C2—C3—H3	118.907	H25B—C25—H25C	109.473
			
C16—N1—C19—C18	0.27 (15)	C1—C2—C7—C10	179.90 (11)
C16—N1—C19—C24	−179.62 (12)	C3—C2—C7—C8	−120.82 (13)
C19—N1—C16—C15	178.62 (12)	C3—C2—C7—C9	117.35 (13)
C19—N1—C16—C17	0.15 (15)	C3—C2—C7—C10	−1.00 (17)
C16—N1—B1—F1	−125.79 (14)	C7—C2—C3—C4	−177.28 (10)
C16—N1—B1—F2	113.99 (15)	C2—C3—C4—C5	−0.29 (19)
C16—N1—B1—N2	−5.49 (19)	C2—C3—C4—C15	179.24 (11)
B1—N1—C16—C15	4.9 (2)	C3—C4—C5—C6	−1.14 (19)
B1—N1—C16—C17	−173.54 (12)	C3—C4—C15—C16	−47.14 (18)
C19—N1—B1—F1	61.66 (19)	C3—C4—C15—C23	133.53 (13)
C19—N1—B1—F2	−58.56 (19)	C5—C4—C15—C16	132.38 (13)
C19—N1—B1—N2	−178.04 (12)	C5—C4—C15—C23	−46.95 (18)
B1—N1—C19—C18	173.90 (12)	C15—C4—C5—C6	179.33 (11)
B1—N1—C19—C24	−6.0 (2)	C4—C5—C6—C1	0.86 (19)
C20—N2—C23—C15	−179.76 (12)	C4—C5—C6—C11	−178.32 (11)
C20—N2—C23—C22	0.77 (15)	C1—C6—C11—C12	59.64 (16)
C23—N2—C20—C21	−0.21 (16)	C1—C6—C11—C13	−63.79 (15)
C23—N2—C20—C25	−179.57 (12)	C1—C6—C11—C14	178.40 (11)
C20—N2—B1—F1	−56.02 (19)	C5—C6—C11—C12	−121.24 (12)
C20—N2—B1—F2	64.34 (18)	C5—C6—C11—C13	115.33 (12)
C20—N2—B1—N1	−176.57 (13)	C5—C6—C11—C14	−2.48 (17)
B1—N2—C20—C21	179.05 (12)	C4—C15—C16—N1	−179.89 (11)
B1—N2—C20—C25	−0.3 (2)	C4—C15—C16—C17	−1.8 (2)
C23—N2—B1—F1	123.11 (13)	C4—C15—C23—N2	177.05 (11)
C23—N2—B1—F2	−116.52 (14)	C4—C15—C23—C22	−3.6 (2)
C23—N2—B1—N1	2.57 (19)	C16—C15—C23—N2	−2.3 (2)
B1—N2—C23—C15	1.0 (2)	C16—C15—C23—C22	177.06 (13)
B1—N2—C23—C22	−178.50 (12)	C23—C15—C16—N1	−0.6 (2)
O1—C1—C2—C3	178.10 (10)	C23—C15—C16—C17	177.49 (13)
O1—C1—C2—C7	−2.77 (17)	N1—C16—C17—C18	−0.52 (16)
O1—C1—C6—C5	−179.44 (10)	C15—C16—C17—C18	−178.77 (14)
O1—C1—C6—C11	−0.28 (19)	C16—C17—C18—C19	0.67 (17)
C2—C1—C6—C5	0.85 (18)	C17—C18—C19—N1	−0.59 (17)
C2—C1—C6—C11	180.00 (11)	C17—C18—C19—C24	179.29 (13)
C6—C1—C2—C3	−2.16 (19)	N2—C20—C21—C22	−0.45 (17)
C6—C1—C2—C7	176.96 (11)	C25—C20—C21—C22	178.88 (14)
C1—C2—C3—C4	1.87 (19)	C20—C21—C22—C23	0.91 (16)
C1—C2—C7—C8	60.08 (16)	C21—C22—C23—N2	−1.04 (16)
C1—C2—C7—C9	−61.75 (16)	C21—C22—C23—C15	179.55 (13)

### Hydrogen-bond geometry (Å, º)

**Table:**

*D*—H···*A*	*D*—H	H···*A*	*D*···*A*	*D*—H···*A*
C12—H12*C*···F2^i^	0.96	2.54	3.4464 (18)	158

Symmetry code: (i) −*x*+1, −*y*+2, −*z*+1.
