# Supplementary figures and images for: Crystal structure of (2-{[3,5-bis­(1,1-di­methyl­eth­yl)-4-hy­droxy­phen­yl](5-methyl-2H-pyrrol-2-yl­idene)meth­yl}-5-methyl-1H-pyrrolido-κ2 N,N′)di­fluoridoboron
Source: Acta Crystallogr E Crystallogr Commun. 2015 Aug 29;71(Pt 9):o694–5. doi: 10.1107/S2056989015015789 (PMC4555381; doi:10.1107/S2056989015015789)

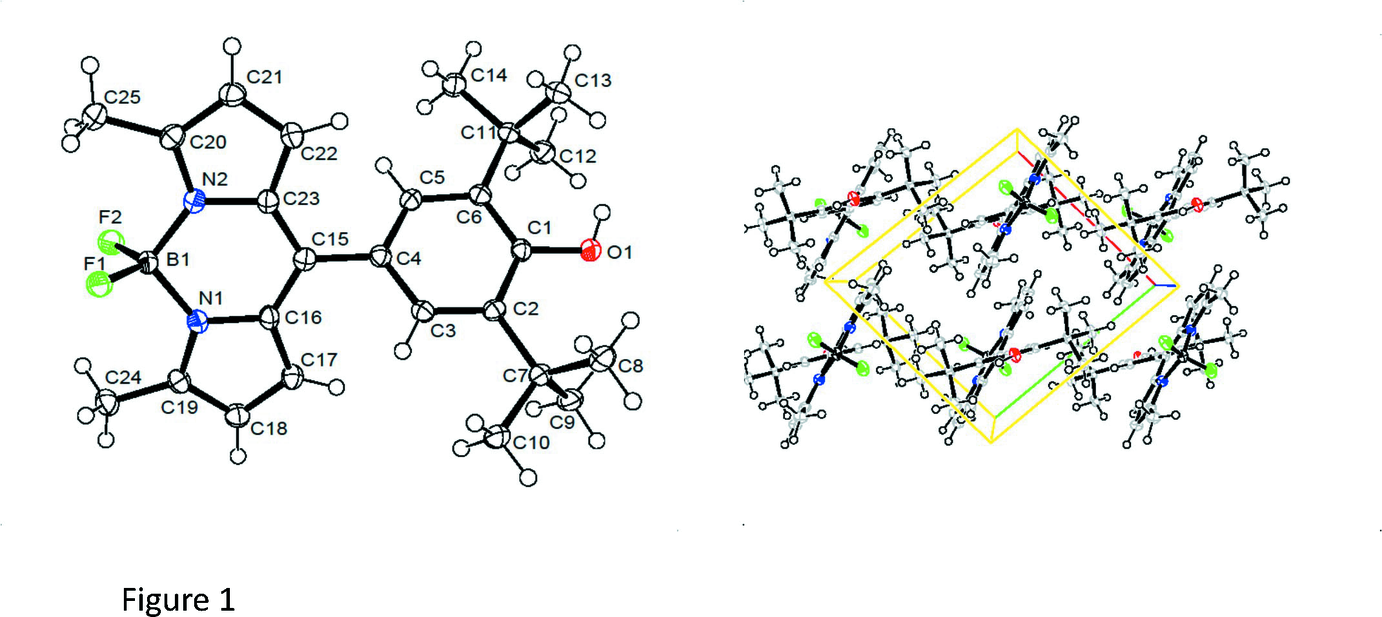

Supplement: Supplementary file 3 [file e-71-0o694-fig1.tif]
